# Monitoring process-related impurities in biologics–host cell protein analysis

**DOI:** 10.1007/s00216-021-03648-2

**Published:** 2021-10-01

**Authors:** Katrine Pilely, Martin Rask Johansen, Rikke Raaen Lund, Thomas Kofoed, Thomas Kjærsgaard Jørgensen, Lars Skriver, Ejvind Mørtz

**Affiliations:** 1 Alphalyse A/S, Odense, Denmark; 2 Savara Aps, Hørsholm, Denmark

**Keywords:** Host cell protein analysis, Process-related impurities, ELISA coverage analysis, Liquid chromatography mass spectrometry, ELISA-MS

## Abstract

During biologics development, manufacturers must demonstrate clearance of host cell impurities and contaminants to ensure drug purity, manufacturing process consistency, and patient safety. Host cell proteins (HCPs) are a major class of process-related impurities and require monitoring and documentation of their presence through development and manufacturing. Even in residual amounts, they are known to affect product quality and efficacy as well as patient safety. HCP analysis using enzyme-linked immunosorbent assay (HCP-ELISA) is the standard technique, due to its simple handling, short analysis time, and high sensitivity for protein impurities. Liquid chromatography mass spectrometry (LC–MS) is an orthogonal method for HCP analysis and is increasingly included in regulatory documentation. LC–MS offers advantages where HCP-ELISA has drawbacks, e.g., the ability to identify and quantify individual HCPs. This article summarizes the available knowledge about monitoring HCPs in biologics and presents the newest trends in HCP analysis with current state-of-the-art HCP measurement tools. Through case studies, we present examples of HCP control strategies that have been used in regulatory license applications, using an MS-based coverage analysis and HCP-ELISA and LC–MS for HCP quantification. This provides novel insight into the rapid evolving strategy of HCP analysis. Improvements in technologies to evaluate HCP-ELISA suitability and the implementation of orthogonal LC–MS methods for HCP analysis are important to rationally manipulate, engineer, and select suitable cell lines and downstream processing steps to limit problematic HCPs.

## Introduction

During biologics development and production, manufacturers must demonstrate clearance of host cell impurities and contaminants to ensure drug purity, manufacturing process consistency, and patient safety [1]. These impurities include host cell DNA and RNA, lipids, aggregates, and proteins expressed endogenously by the host cells termed host cell proteins (HCPs) [2]. HCPs are often proteins associated with vital cell functions, such as cell proliferation, gene transcription, protein synthesis, cell survival, cell growth, etc., and are released during the fermentation process due to cell death and lysis. Residual impurities may affect product quality, efficacy, and safety, which is why the monitoring of HCPs is a critical quality attribute (CQA) in the production of biological drug substances [3, 4]. Anti-HCP enzyme-linked immunosorbent assay (HCP-ELISA) has been the standard method of choice for HCP measurement due to its ability to quickly assess the level of HCP impurities [5]. HCP-ELISA provides a simple immunological measure of the total impurity level expressed as immuno-equivalent nanograms of HCPs per milligram drug substance. However, HCP-ELISA offers no information about the identity and amount of individual HCPs. With the emergence of liquid chromatography mass spectrometry (LC–MS), the possibilities of analyzing and controlling process-related HCP impurities in biologics manufacturing have significantly improved [6]. Setting up a strategy for HCP analysis is a complex undertaking, as HCPs are a complex group of impurities composed of thousands of different proteins present at cell harvest that may co-purify with the drug through downstream processing [1, 7]. Due to the importance of HCP removal, the field of HCP analysis is rapidly evolving in the biopharmaceutical industry. However, many improvements and examples of improved manufacturing are not published in peer-reviewed journals by the manufacturers. In this paper, the available knowledge about HCP analysis and the current state-of-the-art HCP measurement tools, with references to recent conference contributions, and presentations by U.S. Food and Drug Administration (FDA) reviewers as well as the authors’ experience from the HCP analysis of multiple biopharmaceuticals, is summarized. Case studies with examples of HCP control strategies, using an MS-based method for coverage analysis and HCP-ELISA and LC–MS for HCP quantification, are presented. The approaches described in the case studies were used for regulatory license application and provide novel insight into the strategy of HCP analysis.

## HCP detection in biologics

The HCP profile of a bioprocess is very difficult to predict and needs to be monitored closely during process development. The product purification processes must be optimized to consistently remove as many HCPs as feasible, with the goal of making the product as pure as possible [8]. HCPs in biopharmaceutical products have the potential to induce immunogenic effects, e.g., by inducing direct antibody- or receptor-mediated responses resulting in a clinical effect in patients, thus affecting the safety or efficacy of the drug [3, 9, 10]. In addition, HCPs can directly alter the quality of the product itself. Proteolytic HCPs, even in minute quantities, have been shown to cleave the desired protein product over time, reduce biological potency, and influence stability [3, 8, 11].

### Regulatory focus on process-related impurities and the HCP assay

**Fig. 1:**
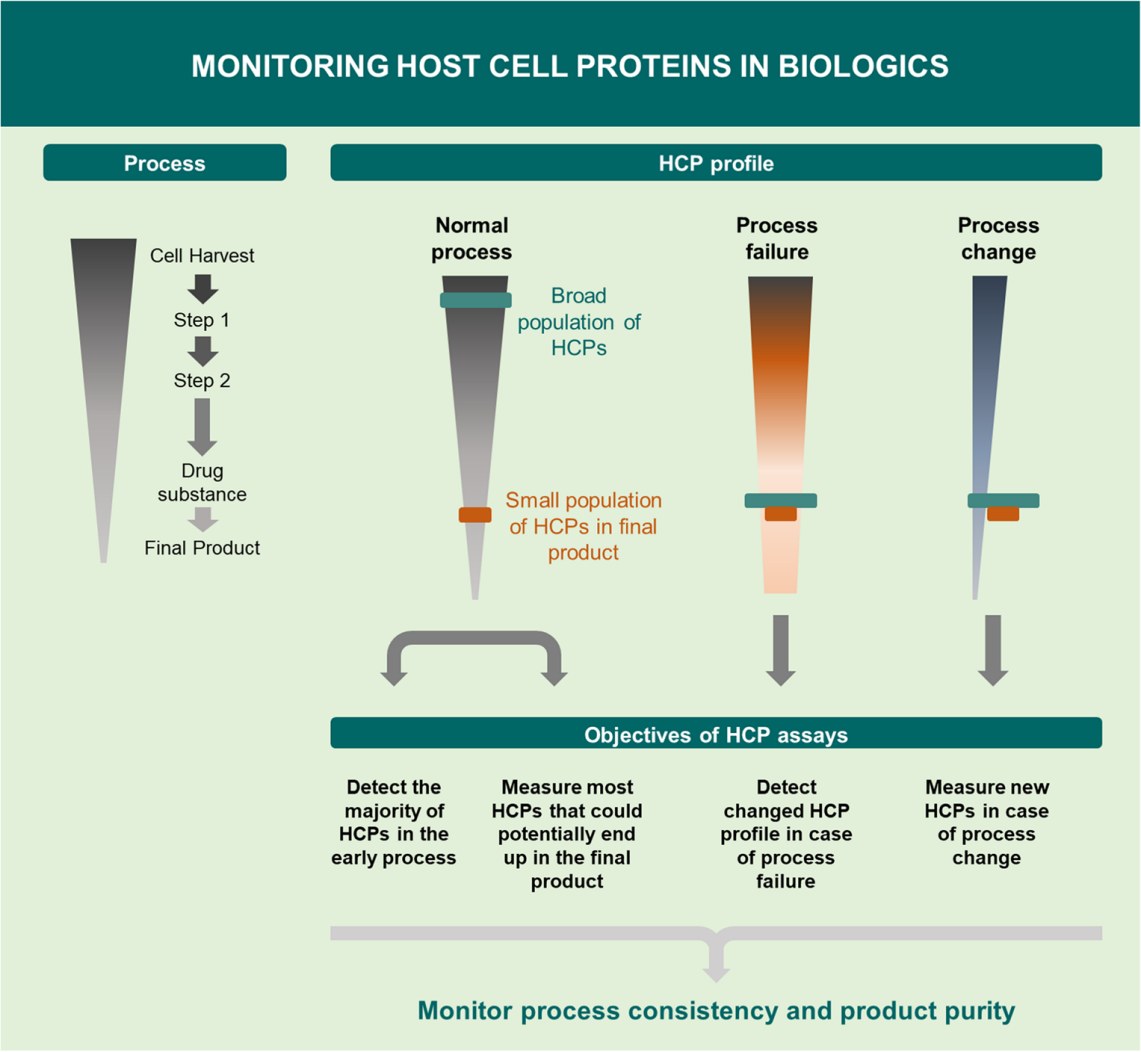
Objectives of HCP assays for monitoring HCPs in biologics. The assay should measure the majority of proteins in the early process, i.e., most of the HCPs that could potentially end up in the final product as well as the small amount of residual HCPs in the purified drug substance [8, 12]. In case of a process failure, e.g., a leaking chromatography column, the HCP assay should be able to detect the changed HCP profile. In case of process change, the chosen HCP assay should be capable of measuring any new HCPs

For years, regulatory authorities have asked for better control of process-related impurities to ensure manufacturing process consistency and patient safety. HCP assays are process consistency tools and should detect the majority of HCPs in the early process, e.g., cell harvest samples, enabling the assay to measure the majority of HCPs that could end up in the purified product. The key objectives of HCP assays in a biopharmaceutical purification process [8, 12] are illustrated in Fig. 1. Manufacturers must demonstrate clearance of HCPs during the purification process. When the manufacturing process is scaled up and the purification process changed during development, the chosen HCP assay should be capable of measuring any new HCPs without requiring the development of a new assay. In the case of a process failure, e.g., a leaking chromatographic column, the HCP assay should be able to detect the changed HCP profile. The HCP assay is also an impurity assay for the purified drug substance and should have a high sensitivity for the low-level HCPs in the final product. Recently, guidance documents have been issued by the US Pharmacopeia (USP) and the European Pharmacopeia on strategies for monitoring HCP impurities [8, 13]. In addition, other guidance documents from the International Council for Harmonization (ICH) are Q6B [4], ICH S6(R1) [14], and ICH Q8(R2) [15]; FDA; European Medicines Agency (EMA); and Chinese Pharmacopeia [16–18]. HCP guidance documents provide no numerical limit of the HCP level as the risk associated with HCP exposure depends on the clinical setting, including dose, route of administration, frequency of exposure, indication, patient population, and the particular impurity [3]. Moreover, a suitable HCP assay should be established early in process development [12]. If the assay has insufficient HCP coverage or does not detect abundant HCPs in the purified product, it may result in project delays at later stages due to assay development and purification improvements required by regulatory authorities [19].

Regulatory focus has pushed the development of improved monitoring methods, and authorities now recommend the use of orthogonal analytical methods, such as LC–MS, electrophoresis, HPLC, and Western blotting, to support the development and validation of the HCP-ELISA, as well as the characterization of process HCPs [8, 13]. In 2020, USP established an expert panel to write a new general chapter on best practices for identification, characterization, and quantitation of HCP impurities in biological products using MS to advice the industry on study designs and analytical standards [20]. Thus, the use and regulatory requirements for HCP analysis by LC–MS are likely to increase in the future.

## Technologies for HCP detection and identification

**Fig. 2:**
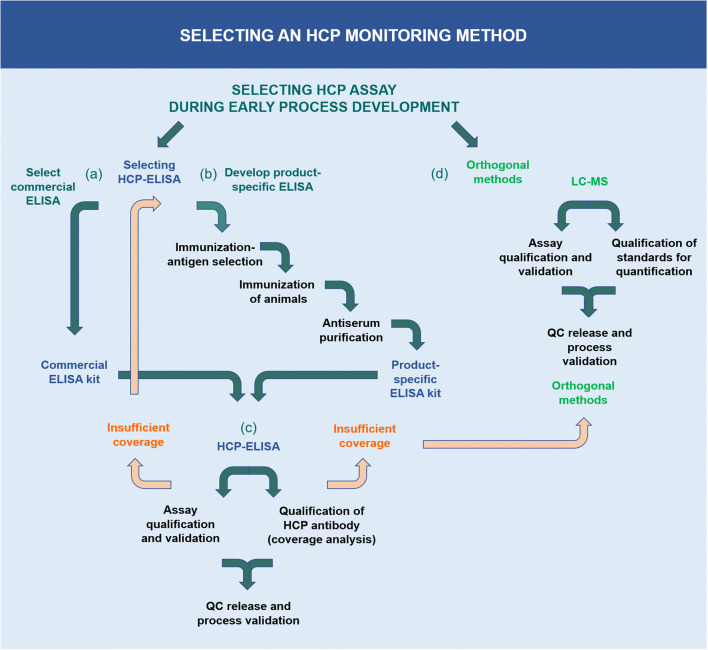
Selecting an HCP monitoring method. (a) Commercial ELISA kits developed from generic strains are widely applied in early drug development stages. Commercial ELISA kits may be used for licensed pharmaceuticals on the market if the assay has been validated with process-specific performance. (b) If no commercial ELISA kit with sufficient HCP coverage is available, it is recommended to develop a product-specific ELISA kit or to use a platform ELISA kit. During the production of the product-specific HCP antibodies, it is important to select and characterize the antigen used for immunization and to consider the species immunized with HCP antigens. (c) Whether using a commercial, platform, or product-specific HCP-ELISA, it is necessary to do a fit-for-purpose validation to the actual process and product, including choosing an HCP standard representative for the samples, checking for quantitative dilutional linearity, and determining sensitivity and the limit of quantification (LOQ) for the drug substance samples, and to characterize the critical reagents: the HCP antigen and the HCP-specific antibodies. The HCP antibody’s ability to cover the HCPs in the process must be evaluated by an HCP coverage analysis. The most common coverage methods are 2D-PAGE and Western blotting; immunoaffinity–chromatography(IAC) and 2D-PAGE (2D-DIGE); and Immunocapture followed by LC–MS. (d) If no HCP-ELISA kit shows sufficient coverage of HCPs, LC–MS is recommended as an orthogonal method to HCP analysis by ELISA. HCP analysis by LC–MS provides identification and quantification of the individual HCPs, as well as the total HCP amount. When using LC–MS for HCP analysis, it is important to do a fit-for-purpose validation of the assay reproducibility, as the analysis is composed of multiple steps and a complex analysis workflow, as well as to qualify the robustness and sensitivity of the chromatography and MS system. This also includes characterization of the critical reagents such as qualification of standard proteins for quantification, i.e., intact proteins spiked-in, in known amounts into each sample

Since HCPs are a highly diverse group of impurities, several technologies and regulatory requirements for HCP analysis exist [1, 7]. Considerations regarding the different technologies for HCP detection, identification, and measurement are described in the sections below and illustrated in Fig. 2.

### Selecting a commercial-, process-, or platform-specific HCP-ELISA

Until recently, ELISA was the only method with sufficient sensitivity to detect the low parts-per-million(ppm) levels of HCP impurities in the presence of milligrams drug substance [5]. HCP analysis by ELISA has several advantages including high availability, throughput, sensitivity, and selectivity. The HCP value obtained with ELISA is the result of animal immunization and antibody reactivity to HCP antigens. It can be considered as an “immunologic equivalent” to the total amount of immunoreactive HCPs and does not equal “molar concentration.” The ELISA value relies on multiple factors, including preparation method of antigens from the host cell line, choice of animal species for immunization, individual animal immune responses, and the HCP standard used in the ELISA for quantification.

Depending on the stage of clinical development, different types of HCP-ELISAs are often applied. Commercial ELISA kits developed from generic strains of, e.g., CHO and *Escherichia coli*, are widely applied in early drug development stages (Fig. 2, (a)), and they may also be used for licensed pharmaceuticals if the assay has been validated with process-specific performance. Commercial HCP-ELISA kits are often utilized because they can be purchased off the shelf and roughly assess the impurity level. Antibodies are raised by several rounds of immunizations in rabbits, goats, sheep, or hens with crude cell lysates of antigens from generic cell lines. In some cases, kits are based on lysates from multiple common strains to measure the HCPs from several different host cell lines. One drawback from using these total protein lysates is the fact that low molecular weight (Mw) proteins often are less immunogenic than higher Mw proteins, resulting in ELISAs with poor detection of low Mw HCPs. Therefore, some commercial kits are prepared by immunizing with high and low Mw protein fractions, ultimately resulting in better detection of small proteins.

For the selection of a suitable HCP-ELISA, several parameters should be considered. The amount of antibody and HCP standard should last the anticipated lifetime of the product. The anti-HCP antibody used in the ELISA is often used for many applications, e.g., as capture and detection antibody in ELISA for process development and batch release and for coverage analysis, as well as for bridging to new reagents. The amount of antibody needed can potentially be a problem when using a commercial ELISA, as vendors do not have an endless stock of the antibody. Eventually, they must produce new antibodies by new rounds of animal immunizations and consequently biopharmaceutical manufacturers are required to do bridging studies and re-validation of GMP release tests. If the expression of the product and the conditions for fermentation and harvest result in a different HCP profile than used to raise the generic commercial ELISA antibody, the HCP coverage may not be sufficient. If no commercial HCP-ELISA has a sufficient coverage, a process- or platform-specific ELISA should be developed (Fig. 2, (b)) [8, 13]. During the production of process-specific HCP antibodies, it is important to carefully select the antigen for immunization and to consider the immunized species.

Whether using a commercial-, platform-, or product-specific HCP-ELISA, it is necessary to do a fit-for-purpose assay qualification and validation to the actual process and product. This procedure includes choosing an HCP standard representative for the samples, checking for quantitative dilutional linearity, determining sensitivity and the limit of quantification (LOQ) for the drug substance samples, and characterizing the ELISA reagents, i.e., the HCP antigen and the HCP-specific antibodies [8] (Fig. 2, (c)). The HCP antigen must be a good representative of the product- or process-specific cell harvest sample to contain most HCPs that could potentially end up in the final product. The HCP-specific antibody must cover a broad spectrum of HCPs potentially found in the drug substance, which is determined by a coverage analysis on an early process sample or mock sample [5, 8, 13, 21]. Other important ELISA parameters are high sensitivity for low-level HCPs in the product and a high specificity to perform well in the presence of high drug protein amounts without cross-reactivity to the drug protein.

### HCP-ELISA coverage analysis methods

ELISA’s ability to cover the HCPs in the process must be evaluated by an HCP coverage analysis. An HCP-ELISA should be capable of detecting, i.e., covering, the majority of HCPs potentially found in process- and final drug samples. The most common coverage methods are 2D-PAGE and Western blotting [8, 21]; immunoaffinity chromatography (IAC) and 2D-PAGE(2D-DIGE) [8, 21]; and immunocapture followed by LC–MS [22, 23]. When using HCP antibody coverage analysis based on gel electrophoresis techniques, the number of gel spots, observed after immunostaining or after immunoaffinity–purification, is compared to the number of spots observed after a total protein stain. These techniques have several limitations resulting in high variability in HCP coverage percentages, such as over-transfer through the membrane or incomplete transfer of HCPs to the blotting membrane; unreliable spot counting with one HCP accounting for several spots; overloading or comigration where one spot may overlay another spot; difficulties comparing spots in blots and gels because of unlike staining methods with different sensitivity; and denaturing conditions during 2D gel electrophoresis destroying native epitopes [8, 21]. In addition, coverage methods relying on immunoaffinity–purification require large amounts of HCP antibody, which might be a limiting factor as the amount of HCP antibody should last the anticipated lifetime of the product [8]. Novel coverage methods, combining immunoaffinity–purification on magnetic beads or in an ELISA plate, with LC–MS, have recently been described [22, 23]. These methods show high sensitivity with identification and quantification of individual HCPs by LC–MS [22, 23]. The challenges to the novel coverage methods relying on LC–MS are that they require sophisticated and expensive mass spectrometry instrumentation, compared to the more commonly used gel and blotting equipment, and may underrepresent some HCPs if they are not digested by trypsin. Insufficient HCP-ELISA coverage may result in the need for orthogonal methods such as LC–MS for HCP analysis, either as the primary HCP assessment tool or as supporting information in addition to an HCP-ELISA [6].

### LC–MS as measurement tool for HCP analysis

Protein separation techniques, such as LC and SDS-PAGE, have been used in combination with MS for proteomics research from the early 1990s, and since 2005, these techniques have been applied for HCP analysis [1, 3–5, 7, 24–28]. Recently, major advances have been accomplished for HCP analysis with faster and more sensitive LC–MS instruments, along with improved public databases containing the protein sequences from expression organisms used in biologics development. By searching the LC–MS data against protein databases containing the amino acid sequences from, e.g., Chinese hamster, *Homo sapiens*, *E. coli*, yeast, insects, rice, etc., it is now possible to identify any HCP from the microorganism or eukaryotic cell line used for biopharmaceutical production.

Regulatory authorities recommend the use of LC–MS as an orthogonal method to ELISA for HCP analysis [8, 12, 13, 19], and since 2017, several biopharmaceutical companies and contract research organizations (CROs) have routinely analyzed HCPs by LC–MS in various protein biopharmaceuticals, including monoclonal antibodies (mAbs), vaccines, and other biologics. Several analysis workflows have proven successful with instruments from different vendors, different database search software, and different quantification methods.

HCP analysis by LC–MS can provide identification and quantification of the individual HCPs, as well as the total HCP amount. The development of sensitive LC–MS methods has added a new dimension to HCP analysis, where each analyte is measured directly in a high-resolution mass detector without relying on anti-HCP antibodies [7, 24–28]. By providing details of each individual HCP, LC–MS significantly improves the possibilities for purification process development to clear the individual HCP and for control of the total HCP content in the purified drug substance [8]. The more detailed HCP information has multiple benefits for biologics development: (A) The exact HCP profile of the purified drug substance can be determined and compared between good manufacturing production (GMP) batches; (B) specific HCPs of potential concern for patient safety and product stability can be identified; and (C) the purification process can be modified to remove specific HCPs of concern, i.e., the most abundant HCPs and potential problematic HCPs. The HCP profile after each purification step enables full control of impurity clearance and demonstrates manufacturing consistency and robustness. In addition, LC–MS allows for protein impurity analysis of complex biologics, such as viral vaccines and gene therapy products, where the process-related impurities arise from multiple organisms and sources, e.g., a human production cell line, recombinant serum albumin, benzonase, and other enzymes used in the manufacturing process [29].

### Choosing an LC–MS HCP analysis method for biologics development

When choosing a suitable LC–MS method for HCP analysis in biologics development, it is necessary to do a fit-for-purpose assay qualification and validation, including qualification of the quantification method (Fig. 2, (d)). HCP analysis by LC–MS is composed of a complex analysis workflow, which is why reproducibility is of key importance. The analysis comprises multiple steps, including sample preparation for denaturation, reduction, alkylation, and tryptic digestion of the proteins into peptides. The peptides are separated by chromatography (HPLC) and analyzed by MS/MS [30]. The samples may contain thousands of digested peptides and result in hundreds of thousands of MS/MS spectra. Integration of the many steps and use of internal quality control (QC) standards are crucial for workflow reproducibility. The robustness and sensitivity of the chromatography and MS system are other essential parameters [8]. For research purposes, proteomics laboratories often use two-dimensional chromatography and nanoflow systems to reach optimal separation power and sensitivity. However, for routine HCP analysis where robustness and throughput are more important, this is not the ideal approach. More robust workflows often apply one-dimensional chromatography with flowrates from 1 to 300 μl per minute, as well as charged surface reversed-phase particle columns with high peptide loading capacity and good separation in mobile phases containing formic acid [31]. The MS data can be acquired in data-dependent mode (DDA) where the instrument selects the peptides for MS/MS fragmentation, or data-independent acquisition mode (DIA) where all peptides are fragmented by MS/MS in sequential mass windows. For reproducible protein identification, it is recommended to require at least 2 peptides per protein and to use updated protein databases from UniProt or NCBI.

### Reproducible quantitation

Quantitative MS methods have been developed in the proteomics field since 1999 where three independent laboratories introduced stabile isotope labelling of peptides and proteins for quantitative analysis of complex protein mixtures [32–34]. Using isotope-label techniques, stabile isotope-labeled peptides are synthesized for target peptides from the proteins of interest. Another approach is label-free techniques where MS signal intensities of peptides are correlated with internal, spiked controls. Silva et al. introduced absolute label-free quantification using the average MS signal of the three most intense peptides from each protein, termed “Hi3” quantification [35], showing a linear correlation to the peptide amount in picomoles [36, 37].

A robust HCP analysis workflow using DIA LC–MS/MS has been developed by the authors of this article [38]. With the DIA analysis, it is possible to acquire MS/MS data on low-abundance HCPs in the presence of large amounts of drug protein. The HCP quantification is based on label-free quantification using the summed MS signal of all the observed peptides from each protein, termed “SumAll” quantitation, showing a linear correlation to the protein amount in nanograms [38]. Intact proteins are spiked-in, in known amounts into each sample, and serve as internal standards for quantification. The protein standards are added before any sample preparation to ensure that the analyte HCPs and the calibration standards undergo identical proteolytic digestion within the same sample matrix. Further, it compensates for any variation in trypsin digestion efficiency and any peptide loss that may occur during sample processing. The workflow includes (A) reproducible and automated sample preparation in a pipetting robot for tryptic digestion and peptide cleanup; (B) robust one-dimensional chromatography system running at 5 μl per minute with a 40-min HPLC gradient; (C) DDA and DIA LC–MS analysis obtaining reproducible data; and (D) data analysis that provides positive identification and reproducible quantification of each HCP.

### Optimization of the purification process by monitoring HCPs and detecting HCPs of concern

The definition of the manufacturing design space for clearance of contaminants in the downstream process is a key requisite for risk assessment. Until recently, the clearance of HCP has been limited to measuring the ELISA value for each purification step, but this provides no guidance for how the purification should be improved. The lack of information about individual HCPs and their amount often leads to cumbersome purification optimization by matrix experiments where the only readout is the ELISA value. With robust LC–MS analysis, the identity and quantification of individual HCPs are obtained in each sample; thus, the HCP profile can be compared between steps and during process development stages. The isoelectric point (pI) and Mw information of the HCPs can be used for a data-driven purification optimization by making simple changes to individual process steps—for example, by changing the pH of an ion exchange buffer or using a different Mw cutoff in size exclusion. Case studies on the use of LC–MS for HCP clearance and process consistency are presented in a later section of this article.

HCPs that are difficult to remove, e.g., hitchhiker HCPs with tight association to the drug protein, may require a re-design of the purification steps. Even the use of gene knock-out expression strains to eliminate unwanted HCPs may be needed, for example, in enzyme replacement therapies, to knock out the hamster homolog of the human drug protein. There are few but distinctive studies of specific HCPs in biopharmaceutical products that have potential adverse effect on patients or on the final product stability. Problematic HCPs were reviewed by Vanderlaan et al. in 2018 [3], and the list continues to grow with more and more products analyzed by LC–MS. Recently, a list of frequently seen problematic/high-risk HCPs was published by the BioPhorum Development Group (BPDG), compiling information about HCPs through literature searches, company experiences, and surveys [39]. Although more information is collected on potential problematic HCPs, there is still not a “safe list” of HCPs, nor a complete list of unwanted HCPs. The problematic HCPs typically fall into the categories of enzymes such as serine protease, disulfide isomerase and phospholipase affecting the stability of the drug product, immunogenic proteins and stimulants of patient immune responses, or abundant hitchhiker proteins binding to the drug protein. Specific HCPs in the drug product may impact drug quality, formulation, biological function, or immunogenicity [40]. A risk assessment tool was published in 2015 by de Zafra et al., enumerating and discussing factors to be considered for residual HCPs identified in the drug product [41]. With the identity of individual HCPs, it is possible to do a risk assessment considering multiple factors such as impact of impurity on activity; safety and immunogenicity; clinical data on anti-drug–antibody formation in patients; prior knowledge on similar drugs and HCPs; dose; route of administration; and severity of disease [41, 42].

## Case studies: HCP analysis by ELISA and orthogonal LC–MS methods in biologics

### Selecting a commercial ELISA kit for HCP analysis by MS-based coverage analysis

For some biopharmaceuticals, it is not possible to develop a product-specific ELISA. For other products, the project timelines may not allow 1–2 years of development of a product-specific ELISA, e.g., for a COVID-19 vaccine. Here, a case study for a drug protein expressed in *E. coli* inclusion bodies is presented. As the drug protein was expressed in *E. coli* inclusion bodies, it was not possible to prepare an immunization antigen without the drug protein to raise the ELISA antibodies. The project aim was to evaluate if one of three available commercial ELISAs (ELISA A, B, and C) had sufficient coverage of the HCPs present in an early process sample to be used as a drug release assay during manufacturing. The HCP coverage analysis was performed by ELISA–MS, and for the assay with the highest coverage, coverage analysis was also performed by classical 2D-PAGE and Western blotting. ELISA–MS is a novel coverage analysis described by Pilely et al. [23], allowing fast screening of commercially available as well as platform- and process-specific ELISAs. In the ELISA–MS coverage analysis, the number of HCPs identified by LC–MS/MS after immunocapture with the HCP antibody is compared to the total number of HCPs identified by LC–MS/MS in the early process sample. In the coverage analysis by 2D-PAGE and Western blotting, the number of gel spots observed after immunostaining (Western blotting) is compared to the number of gel spots observed after a total protein stain (2D-PAGE).

In the case study, a relatively high coverage was obtained for the three commercial HCP-ELISA antibodies. The HCP antibody from ELISA A showed the highest coverage with 952 individual HCPs out of the 1265 HCPs identified in the early process sample, corresponding to a coverage of 75%. The HCP antibody from ELISA B and C covered 917 and 662 HCPs out of 1265 HCPs, respectively. A coverage analysis of the HCP antibody from ELISA A, performed by classical 2D-PAGE and Western blotting, resulted in a coverage of 62% with 238 out of 383 HCP-related protein gel spots. This shows several advantages of utilizing LC–MS methods for coverage analysis. Compared to coverage analysis by 2D-PAGE and Western blotting, ELISA–MS has a high resolution demonstrated by the high number of identified HCPs in the antigen samples, i.e., 1265 named proteins versus 383 observed gel spots. In addition, ELISA–MS enables tight control of nonspecific binding through experiments using control antibodies. The antibody coverage of HCP impurities in the drug substance was determined by comparing the list of HCPs identified by LC–MS/MS in the purified drug substance with the list of HCPs covered by the different HCP antibodies from the commercial ELISA kits. ELISA A showed the highest coverage by detecting nine out of ten HCPs present in the purified drug substance. ELISA B and ELISA C showed a coverage of eight and six out of ten of the HCPs in the purified drug substance, respectively. In this evaluation of commercial ELISAs, the HCP antibody from ELISA A had the highest overall coverage for the specific bioprocess as well as the highest coverage of the HCPs present in the purified product. The results demonstrate the suitability of commercially available ELISA reagents for HCP measurements, making it acceptable for HCP surveillance for this specific bioprocess. The results also show the advantages of MS providing the identity of each covered protein, allowing evaluation of protein-specific coverage, with a much higher resolution power than classical 2D-PAGE methods.

### HCP–ELISA and LC–MS for control of drug substance purity and risk assessment

The total HCP content in drug substances should be monitored with a validated method for GMP release testing. Table 1 shows the HCP measurement of seven drug substance GMP batches by three analysis methods: a generic commercial ELISA, a platform ELISA, and LC–MS. The generic commercial ELISA kit was used for release of early-stage drug substance batches. At a later stage of process development, the generic ELISA was replaced with a platform-specific ELISA developed exclusively on *E. coli* proteins from the same strain and protein extraction method as used in the manufacturing process, leading to the quantitation of significantly higher amounts of HCPs. In parallel, all batches were analyzed by LC–MS. The proteins were identified and quantified by a minimum two peptides, and the MS signal was calculated with SumAll quantification [38]. Although all three analysis methods in Table 1 measure the total HCP content in the unit nanograms per milligram drug substance (ppm), the numbers vary because the analysis principles are different. An ELISA gives a semi-quantitative measure of the total HCP content of a sample determined as immuno-equivalent nanograms HCP per milligram drug substance. The quantification is semi-quantitative as the quantification by ELISA depends on the reactivity and coverage of the polyclonal HCP antibody used to capture the HCPs and the reference antigen used as standard for quantification. The commercial ELISA, described in Table 1, has a low HCP coverage and uses the generic HCP standard with the kit, resulting in the underestimation of the HCP content of the sample. The platform ELISA covers the majority of HCPs (75% coverage) and uses a process-specific HCP standard. The LC–MS method measures all HCPs quantifiable with at least two peptides and uses internal intact protein standards for quantitation. The results in Table 1 show that the techniques are complementary but the results are not directly identical. This highlights the benefits of using both a suitable HCP-ELISA and LC–MS for HCP analysis, and shows the importance of selecting a suitable HCP ELISA as early in the process development as possible, preferably before or as part of clinical phase I.

**Table 1:** Results from a generic commercial ELISA, a validated platform ELISA, and LC–MS analysis of seven drug substance GMP batches. The total HCP content is measured in parts per million (nanograms (ng) HCP per milligram (mg) drug substance)

**Total HCP amount (ppm, ng/mg)**			
**Batch ID**	**Generic commercial ELISA**	**Platform ELISA**	**LC-MS**
1	10	123	236
2	5	32	182
3	4	26	282
4	5	116	392
5		171	439
6		229	352
7		211	345

HCP analysis by LC–MS was, in this case, implemented during early process development, and the results from HCP analysis of a six-step purification process demonstrate the high level of information gained by LC–MS. Table 2 presents an overview of the HCP levels in each purification step and how the manufacturing process clears each individual HCP. The total level of HCP decreases from 193,169 ppm (ng HCP/mg drug protein) in the step one sample to 401 ppm in the step six sample.

**Table 2:** HCP clearance throughout a purification process from early process development. Quantitative MS analysis on each of the six major unit operations in a biopharmaceutical process. The HCPs are quantified individually, and the sum calculated in parts per million (nanograms (ng) HCP per milligram (mg) drug substance). Theoretical molecular weight (Mw, Dalton) and pI are calculated from the database amino acid sequence. Adopted from https://www.alphalyse.com/wp-content/uploads/2015/09/HCP-analysis-by-LC-MS-1.pdf

	**Purification steps**								
**Host cell protein**	**Step 1**	**Step 2**	**Step 3**	**Step 4**	**Step 5**	**Step 6**	**Mw**	**pI**	**Protein name**
sp|P0C058|IBPB_ECOLI	4274	2905	2154	186	229	111	16,093	5.2	Small heat shock protein IbpB
sp|P0A9A9|FUR_ECOLI	158	284	296	142	147	94	16,795	5.7	Ferric uptake regulation protein
sp|P0ABK5|CYSK_ECOLI	597	913	711	618	200	68	34,490	5.8	Cysteine synthase A
sp|P69783|PTGA_ECOLI	33	250	378	256	185	62	18,251	4.7	PTS system glucose-specific EIIA component
sp|P0A8J4|YBED_ECOLI	432	215	253	222	112	21	9827	5.5	UPF0250 protein YbeD
sp|P02930|TOLC_ECOLI	41	283	187	417	57	11	53,741	5.2	Outer membrane protein TolC
sp|P62623|ISPH_ECOLI	312	1146	855	231	62	15	34,775	5.2	4-Hydroxy-3-methylbut-2-enyl diphosphate reductase
sp|P0ADP9|YIHD_ECOLI	33	28	32	11	10	18	10,275	5.1	Protein YihD
sp|P0A763|NDK_ECOLI	106	240	100	349	113		15,463	5.6	Nucleoside diphosphate kinase
sp|P35340|AHPF_ECOLI	67	291	171	174	48		56,177	5.5	Alkyl hydroperoxide reductase subunit F
sp|P0A717|RIBA_ECOLI	345	297	106	48	45		21,836	5.6	GTP cyclohydrolase-2
sp|P08200|IDH_ECOLI	390	271	166	355	42		45,757	5.2	Isocitrate dehydrogenase
sp|P0AEN1|FRE_ECOLI	741	870	849	1404	33		26,242	5.3	NAD(P)H-flavin reductase
sp|P69797|PTNAB_ECOLI	284	339	240	25	26		35,048	5.7	PTS system mannose-specific EIIAB component
sp|P0ADE8|YGFZ_ECOLI	18	113	45	150	26		36,094	5.2	tRNA-modifying protein YgfZ
sp|P0A6K3|DEF_ECOLI	129	136	55	61	26		19,328	5.2	Peptide deformylase
sp|P0AB91|AROG_ECOLI	56	231	188	42	22		38,010	6.1	Phospho-2-dehydro-3-deoxyheptonate aldolase
sp|P0ABP8|DEOD_ECOLI	33	113	78	19	20		25,950	5.4	Purine nucleoside phosphorylase DeoD-type
sp|P0A825|GLYA_ECOLI	128	332	204	263	13		45,317	6.0	Serine hydroxymethyltransferase
sp|P36683|ACNB_ECOLI	129	87	82	9	8		93,498	5.2	Aconitate hydratase B
Number of HCPs	562	245	206	67	25	8			
Total HCP ppm	193,169	48,548	33,391	9599	1493	401			
**HCP % (w/w)**	**19.32%**	**4.85%**	**3.34%**	**0.96%**	**0.15%**	**0.04%**			

**Fig. 3:**
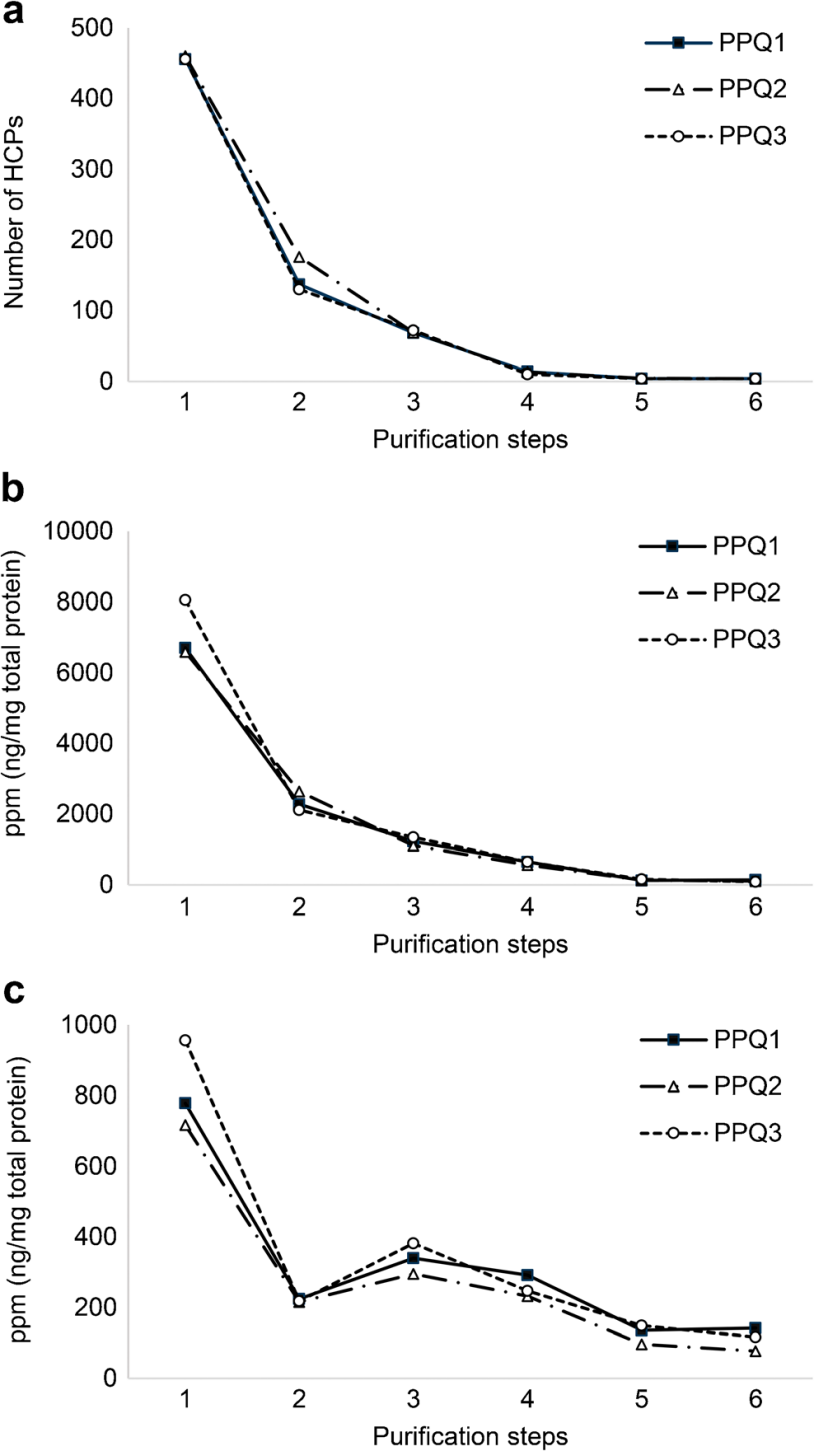
HCP clearance during the downstream manufacturing process involving six purification steps. The process HCP data are shown for three PPQ batches, PPQ1–3. **a** The total number of HCPs throughout the process. **b** and **c** Clearance of two major HCPs, showing their amount in parts per million (nanograms (ng) HCP per milligram (mg) total protein). Note the difference in the scales

Later in the product development, the strategy of performing both LC–MS and HCP-ELISA was also applied for three PPQ batches to verify the process robustness and consistency of the manufacturing process to be used for commercial drug substance production. Figure 3a shows the clearance of HCPs during three consecutive PPQ batches and highlights the process performance and consistency in reducing HCPs throughout the process. Four HCPs contributed to approximately 80% of the total HCP amount in the drug substance and could be monitored individually in the three PPQ batches. The reduction of two of the four specific HCPs during the purification process is illustrated in Fig. 3b, c. The three PPQ batches are highly consistent with regard to the most abundant HCPs, and the process effectively reduces the level of HCP to an acceptable level. The most abundant HCP is present in all batches and is significantly cleared in the purification process from a level of > 8000 to 89 ppm in the drug substance (Fig. 3b). These examples illustrate how the quantitative HCP analysis by LC–MS can assist process development, eliminate unwanted HCPs, and be used as a tool for HCP monitoring and clearance. The HCP control strategy with HCP-ELISA and LC–MS was used for a regulatory license application.

## Outlook and conclusions

Ideally, HCP assays should have the capability to detect all HCPs quantitatively in a high-throughput manner. However, due to the complexity and number of potential HCPs that could end up in the purified drug product, it is not yet possible to fulfill these requirements with one single technology. Traditional HCP analysis by ELISA is an important tool for high-sample-throughput monitoring of immunoreactive HCPs. HCP-ELISA has a high sensitivity, with a limit of detection of around 1 ppm; is relatively easy to perform in standard analytical labs; and can be validated for GMP release testing. The major drawback of HCP-ELISA is the lack of identification and quantification of individual HCPs and the risk of missing non-immunoreactive HCPs. The quantitative LC–MS analysis methods enables identification of HCPs of potential concern and detection of HCPs not covered by the HCP-ELISA. The combination of HCP analysis methods allows for a highly sensitive evaluation of product purity during development and an increase of process knowledge. This enables process developers to improve and assess the process robustness to provide consistent products for the patients and perform risk assessment associated with product quality according to regulatory guidelines. A detailed MS-based HCP coverage analysis and direct identification and quantification of individual HCPs by LC–MS enable an HCP surveillance strategy based on ELISA technology combined with orthogonal LC–MS analysis. The improved HCP surveillance will result in a final HCP profile with the lowest achievable risk, which is beneficial to both the pharmaceutical industry and patient safety.

## Data Availability

Not applicable.
